# Evaluation of inflammation indices according to the Parkland grading scale in laparoscopic cholecystectomy: can inflammation indices predict operative difficulty? A retrospective cross-sectional study

**DOI:** 10.1186/s12893-026-03934-9

**Published:** 2026-06-17

**Authors:** Yavuz Selim Kahraman, Veysel Garani Soylu, Öztürk Taşkın, Ufuk Demir

**Affiliations:** 1 Department of General Surgery, Division of Surgical Oncology, Kastamonu Training and Research Hospital, Kuzeykent Neighborhood, Cankat Street No: 4, Kastamonu, 37150 Türkiye; 2 Department of Intensive Care, Faculty of Medicine, Kastamonu University, Kastamonu, Türkiye; 3 Department of Anesthesiology and Reanimation, Faculty of Medicine, Kastamonu University, Kastamonu, Türkiye

**Keywords:** Parkland Grading Scale, Operative Difficulty, Inflammatory Burden Index, Systemic Inflammation Response Index, Systemic Immune-Inflammation Index

## Abstract

**Background:**

The Parkland Grading Scale (PGS) is a practical intraoperative grading system used to assess operative difficulty during laparoscopic cholecystectomy. This study aimed to investigate the relationship between PGS scores and preoperative inflammatory indices and to evaluate whether these indices were associated with operative severity and intraoperative complexity.

**Methods:**

This retrospective cross-sectional study included 1054 patients who underwent elective laparoscopic cholecystectomy for gallstone disease between January 2018 and January 2025. Demographic characteristics, laboratory parameters, inflammatory indices, operative duration, conversion rates, and PGS scores were analyzed. Univariate and multivariate logistic regression analyses were performed to identify variables independently associated with higher PGS severity. Receiver operating characteristic (ROC) curve analysis was used to determine diagnostic performance.

**Results:**

According to the PGS classification, 451 patients were classified as PGS 1, 146 as PGS 2, 213 as PGS 3, 164 as PGS 4, and 80 as PGS 5. Operative duration and conversion from laparoscopic to open cholecystectomy increased significantly with increasing PGS scores (*p* < 0.001). In multivariate logistic regression analysis, type 2 diabetes mellitus (T2DM), CRP/albumin ratio (CAR), neutrophil/lymphocyte ratio (NLR), and inflammatory burden index (IBI) were identified as independent predictors of higher PGS scores (all *p* < 0.001). ROC analysis demonstrated that IBI had the highest discriminatory performance (AUC: 0.792), followed by NLR (AUC: 0.766) and CAR (AUC: 0.764).

**Conclusions:**

Preoperative inflammatory indices, particularly IBI, NLR, and CAR, were significantly associated with higher PGS scores and increased operative difficulty in laparoscopic cholecystectomy. These markers may help identify patients with increased operative complexity and improve preoperative surgical planning.

## Background

The difficulty of surgery during laparoscopic cholecystectomy varies considerably depending on the degree of gallbladder inflammation, fibrosis, adhesions, and anatomical disruption [1, 2]. Accurate assessment of surgical difficulty is clinically important for surgical planning, prevention of complications, and evaluation of conversion risk [3].

In recent years, various inflammatory biomarkers derived from routine blood parameters have been investigated as potential indicators of inflammatory severity and surgical complexity. There is a positive correlation between gallstone incidence and high levels of inflammatory markers [4]. The severity of inflammation can be assessed using simple blood parameters. One of the inflammation parameters, the inflammatory burden index (IBI), is an inflammatory marker calculated from C-reactive protein (CRP), neutrophil, and lymphocyte levels and is associated with the prognosis of various tumours [5]. In particular, the IBI has been reported to be the most accurate index for predicting the prognosis of patients with colorectal cancer [6]. Another inflammation-related index is the systemic inflammatory response index (SIRI). The SIRI is calculated from the levels of neutrophils, monocytes and lymphocytes [7]. The SIRI can predict the development and severity of acute pancreatitis and may serve as an independent prognostic factor in patients with hepatocellular carcinoma. Positive correlations have also been reported between the SIRI and cardiovascular and all-cause mortality in the American population [8–10]. The systemic immune-inflammation index (SII) is an inflammatory marker calculated from neutrophil, platelet and lymphocyte counts [11]. The SII is a biomarker used in malignancies and inflammatory diseases and has been reported to be a valuable prognostic factor in conditions such as hepatocellular carcinoma, cervical cancer and small cell carcinoma [12].

Laparoscopic cholecystectomy for gallstones can be a very simple procedure or a difficult operation that can last for hours and cause serious complications. The degree of operative difficulty is quite variable. Numerous studies have been conducted to predict the operative difficulty in laparoscopic cholecystectomy [13]. An important tool used in these studies is the Parkland grading scale (PGS). The Parkland grading scale is an intraoperative system used to classify the severity of gallbladder disease on the basis of inflammation and gallbladder anatomy. It is easy to apply and can reveal gallbladder complexity in a simple and reliable manner, aiding in the prediction of surgery difficulty [14, 15].

This study aimed to investigate the association between the Parkland Grading Scale and preoperative inflammatory indices in patients undergoing laparoscopic cholecystectomy and to evaluate whether these indices were related to operative severity and intraoperative complexity.

## Methods

### Study design and ethical approval

This was a single-centre retrospective cross-sectional study. The study was conducted at Kastamonu Training and Research Hospital. Ethical approval for the study was granted by the Kastamonu University Faculty of Medicine Non-Interventional Clinical Research Ethics Committee (Decision number: 2025 − 100, Date: 18.12.2025). The study was conducted in accordance with the principles of the Declaration of Helsinki (1964). Given that this was a retrospective study based on existing data, informed consent was not needed. The study was conducted at Kastamonu Education and Research Hospital between January 2018 and January 2025 and included patients aged 18 years and older who underwent elective laparoscopic cholecystectomy surgery for gallstones.

### Patient selection and inclusion/exclusion criteria

The study included all adult patients aged 18 years and older who underwent elective laparoscopic cholecystectomy for gallstone disease during the study period, with no upper age limit applied. Only elective laparoscopic cholecystectomy cases performed under stabilized clinical conditions were included. Although inflammatory markers may decrease in elective settings, chronic inflammation, fibrosis, adhesions, and recurrent biliary attacks may still contribute to higher PGS severity and operative complexity. Emergency cholecystectomy cases for active severe acute cholecystitis were excluded. Patients who underwent primary open cholecystectomy without laparoscopic initiation were excluded from the study because the Parkland Grading Scale is an intraoperative grading system specifically designed for laparoscopic cholecystectomy. Other exclusion criteria for the study included the following: continuous use of anti-inflammatory medications within the past six months, incomplete clinical or laboratory data, ongoing chemotherapy or radiotherapy, and known haematological or systemic immunological diseases. Patients presenting with acute cholecystitis were initially managed according to institutional conservative treatment protocols, including intravenous fluids, analgesia, and antibiotic therapy when clinically indicated. Delayed elective laparoscopic cholecystectomy after clinical stabilization was included in the study, whereas emergency cholecystectomy cases were excluded. Patient management was performed in accordance with contemporary guideline-based approaches, including the Tokyo Guidelines.

The study focused exclusively on elective laparoscopic cholecystectomy cases in order to evaluate the relationship between chronic inflammatory burden and operative difficulty under relatively standardized surgical conditions. Although acute inflammatory markers may normalize in elective settings, persistent subclinical inflammation, fibrosis, adhesions, and recurrent gallbladder attacks may still contribute to operative complexity and higher PGS scores. Patients undergoing emergency cholecystectomy for acute severe cholecystitis were not included. Delayed cholecystectomies performed after initial conservative management were included only if patients underwent surgery under elective clinical conditions after stabilization.

### Data collection

Retrospective file review and hospital information system data on patient age, sex, diabetes mellitus status, previous hospital visits because of gallstones, haematological blood parameters within 24 h prior to surgery, type of surgery (entirely laparoscopic or laparoscopic initiation with conversion to conventional surgery) and surgery duration (from the first incision to skin closure/minutes) were recorded.

Patients’ Parkland Grading Scale (PGS) classifications were made from the surgical notes and recorded.All laboratory blood parameters and inflammatory indices used in the study were calculated from blood samples obtained within 24 h prior to surgery as part of the routine preoperative evaluation (reviewer 4 − 1). Patients’ Parkland Grading Scale (PGS) classifications were retrospectively determined from detailed operative reports documented immediately after surgery.

PGS was developed to assess the severity of gallbladder disease during laparoscopic cholecystectomy. The scale is classified from 1 to 5 based on degree of inflammation and increased surgical difficulty [14]. A prospective validation study demonstrated that the PGS is a valid and reliable grading system associated with laparoscopic cholecystectomy outcomes, with an Intraclass Correlation Coefficient (ICC) of 0.821 [15] Prospective validation study has reported that The PGS is a valid and reliable scale for predicting laparoscopic cholecystectomy outcomes. A prospective validation study showed that the PGS, with an Intraclass Correlation Coefficient (ICC) of 0.821, has high reliability [15]. The PGS is shown in Table 1 [13]. The composite inflammatory biomarkers were calculated using the following formulas:

- Inflammatory burden index (IBI) = CRP × neutrophils/lymphocytes
- Systemic Inflammation Response Index (SIRI) = neutrophils × monocytes/lymphocytes
- Systemic immune-inflammation index (SII) = neutrophils × platelets/lymphocytes [16, 17]

**Table 1:** Parkland grading scale [14, 15]

PGS	Explanation
1	Normal appearing gallbladder (“robin’s egg blue”) No adhesions present Completely normal gallbladder
2	Minor adhesions at neck, otherwise normal gallbladder Adhesions restricted to the neck or lower of the gallbladder
3	Presence of ANY of the following: Hyperaemia, pericholecystic fluid, adhesions to the body, distended gallbladder
4	Presence of ANY of the following: Adhesions obscuring majority of gallbladder Grade I-III with abnormal liver anatomy, intrahepatic gallbladder, or impacted stone (Mirizzi)
5	Presence of ANY of the following: Perforation, necrosis, inability to visualize the gallbladder due to adhesions

### Statistical analysis

Statistical analysis of the data was performed using IBM SPSS Statistics version 26 (IBM Corp., Armonk, NY, USA). The distribution of continuous variables was evaluated using visual (histogram) and analytical methods. Data not following a normal distribution are presented as the median (interquartile range, IQR), whereas categorical variables are presented as counts and percentages (%). For intergroup comparisons according to the Parkland grading scale scores, the Kruskal–Wallis test was used for continuous variables, and the chi-square test was used for categorical variables. Where deemed necessary, pairwise comparisons were performed for variables found to be significant. A p value < 0.05 was considered to indicate statistical significance. In the logistic regression analysis, the variables were divided into two groups the basis of the Parkland grading scale scores: Parkland 1 and Parkland ≥ 2. An additional subgroup analysis comparing PGS 1–3 and PGS 4–5 was performed to evaluate factors associated with high-grade operative severity. First, univariate logistic regression analysis was performed to examine the relationship of each variable with a Parkland score ≥ 2, and the results are reported as odds ratios (ORs) and 95% confidence intervals (CIs). Parameters found to be significant in the univariate analysis were included in the multivariate logistic regression model to determine independent predictors. The results are presented as adjusted odds ratios (ORs) and 95% CIs. Receiver operating characteristic (ROC) curve analysis was performed to evaluate the diagnostic performance of the independent predictors. The area under the curve (AUC) value, optimal threshold value (Youden index), sensitivity, and specificity were calculated. In all analyses, a two-tailed p value < 0.05 was considered to indicate statistical significance.

## Results

**Fig. 1:**
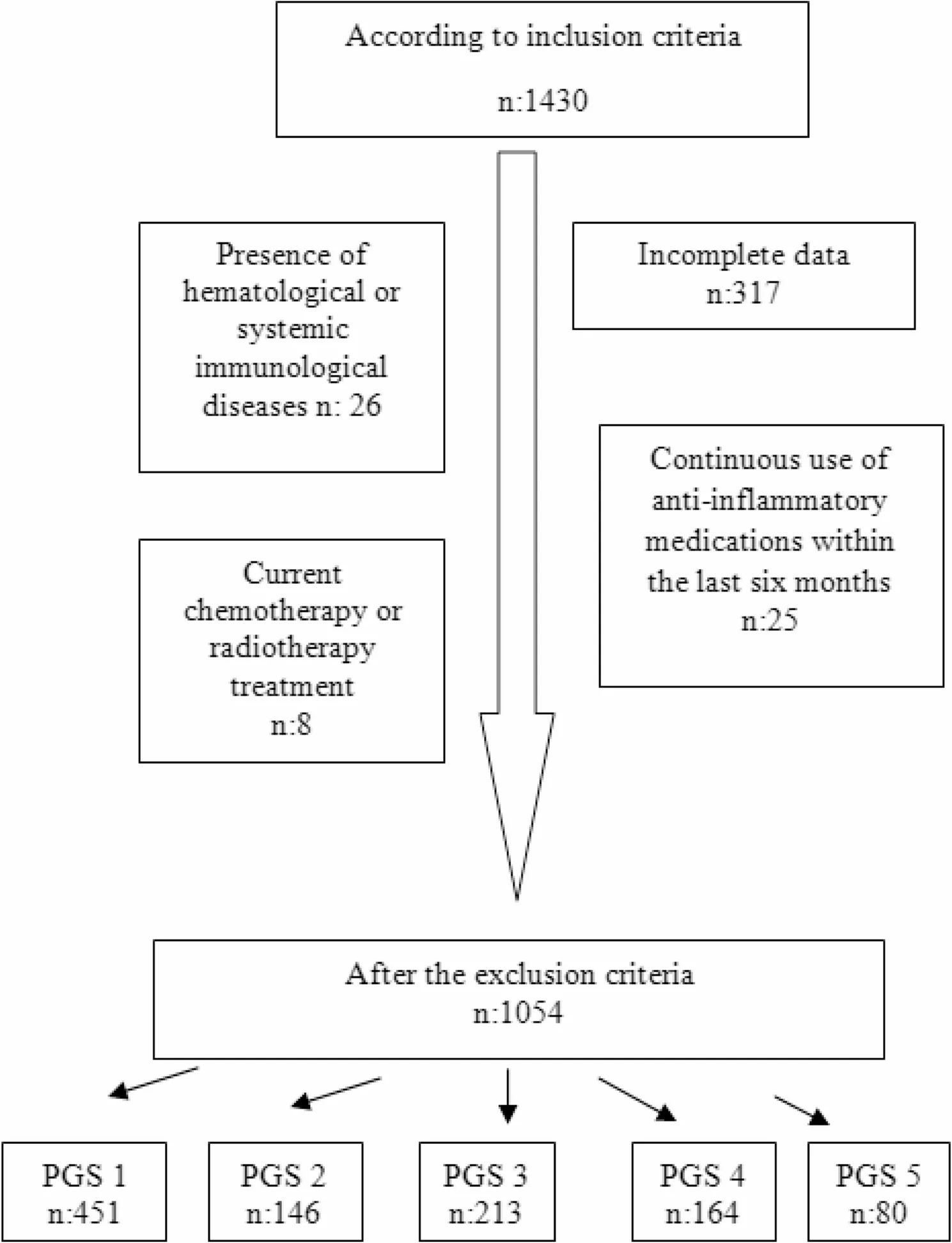
Inclusion and exclusion criteria for the study and flow chart

In accordance with the study’s exclusion criteria, 376 patients were excluded, and 1,054 were ultimately included. According to the Parkland grading scale, of the 1054 patients, 451 were in the PGS 1 group, 146 were in the PGS 2 group, 213 were in the PGS 3 group, 164 were in the PGS 4 group, and 80 were in the PGS 5 group (Fig. 1). The study included 1054 patients who underwent elective laparoscopic cholecystectomy for the treatment of gallstones. The median patient age was 53 years. A total of 322 (30.6%) patients were male, and 185 (17.6%) patients had T2DM. The median duration of surgery was 52 min. In 164 (15.6%) patients, laparoscopic cholecystectomy was converted to conventional open cholecystectomy. A total of 724 (68.6%) patients had been admitted to the hospital for the first time because of gallstone symptoms. The demographic characteristics, laboratory findings, and inflammatory indices of all patients included in the study are summarized in Table 2.

**Table 2:** Demographic, clinical and laboratory characteristics of all patients

Variables	Value[Median(Q1-Q3) or *n*/%]
Age, year	53 (42–63)
Sex (male) (n/%)	322 (30.6%)
T2DM (n/%)	185 (17.6%)
Previous hospital visit due to gallstone symptoms(n/%)	
One visit(n/%)	724(68.6%)
Two visits(n/%)	293(27.7%)
Three visits(n/%)	37(3.7%)
Surgery duration(minutes)	52 (40–63)
Laparoscopic initiation with conversion to conventional surgery(n/%)	164(15.6%)
WBC count (10^3^/µl)	8.90 (6.90–11.40)
CRP level (mg/L)	18.00 (7.00–55.00)
Neutrophils count (10^3^/µl)	5.90 (4.10–9.10)
Platelet count (10^3^/µl)	259 (210–320)
Lymphocyte count (10^3^/µl)	1.70 (1.20–2.40)
Monocyte count (10^3^/µl)	0.60 (0.45–0.78)
Albumin level (g/dL)	4.00 (3.60–4.30)
AST level (U/L)	28 (20–45)
ALT level (U/L)	30 (18–55)
Total bilirubin level (mg/dL)	0.80 (0.50–1.20)
Direct bilirubin level (mg/dL)	0.20 (0.10–0.40)
ALP level (U/L)	95 (70–140)
GGT level (U/L)	45 (25–90)
Neutrophil/lymphocyte ratio (NLR)	3.30 (1.90–8.10)
Monocyte/lymphocyte ratio (MLR)	0.36 (0.23–0.67)
Platelet/lymphocyte ratio (PLR)	155 (110–225)
CRP/albumin ratio (CAR)	9.50 (3.80–28.00)
Inflammatory Burden Index (IBI)	55 (15–180)
Systemic Inflammation Response Index (SIRI)	2.10 (1.00–5.60)
Systemic Immune-Inflammation Index (SII)	940 (500–2100)

*WBC* White blood cell, *CRP* C-reactive protein, *AST* Aspartate aminotransferase, *ALT* Alanine aminotransferase, *ALP* Alkaline phosphatase, *GGT* Gamma-glutamyl transferase

Reference laboratory ranges: WBC: 4–10 ×10³/µL, CRP: 0–5 mg/L, neutrophils: 2–7 ×10³/µL, lymphocytes: 1–3 ×10³/µL, monocytes: 0.2–0.8 ×10³/µL, platelet count: 150–400 ×10³/µL, albumin: 3.5–5.0 g/dL, AST: 5–40 U/L, ALT: 5–41 U/L, total bilirubin: 0.2–1.2 mg/dL, direct bilirubin: 0–0.3 mg/dL, ALP: 40–130 U/L, GGT: 8–61 U/L

According to the Parkland grading scale, 451 patients were classified as PGS 1, 146 as PGS 2, 213 as PGS 3, 164 as PGS 4, and 80 as PGS 5 (Fig. 1). Recurrent hospital admissions because of gallstone symptoms were more frequent in patients with higher PGS grades. Among patients with three previous hospital visits due to gallstone symptoms, 14 (38%) were in the PGS 4–5 group. These findings suggest that recurrent hospital admissions because of gallstone symptoms may be associated with increased operative complexity and higher PGS severity. T2DM was significantly associated with increasing PGS severity (*p* < 0.001). Operative duration also differed significantly among the groups (*p* < 0.001). The median operative duration was longest in the PGS 5 group (63 min). Conversion from laparoscopic to open cholecystectomy was most frequent in the PGS 4 and PGS 5 groups. In the PGS 5 group, all 80 (100%) patients underwent conversion to open surgery, whereas 40 (24%) patients in the PGS 4 group required conversion. These findings were statistically significant according to PGS severity (*p* < 0.001). The inflammatory indices IBI, SIRI, and SII differed significantly among the PGS groups (*p* < 0.001 for all comparisons). Increasing PGS severity was associated with progressively higher inflammatory index values (Table 3).

**Table 3:** Demographic, laboratory and operative parameters according to the Parkland grading scale

Variables	PGS 1(*n* = 451) Value [Median (Q1–Q3) or *n* (%)]	PGS 2 (*n* = 146) Value [Median (Q1–Q3) or *n* (%)]	PGS 3 (*n* = 213) Value [Median (Q1–Q3) or *n* (%)]	PGS 4 (*n* = 164) Value [Median (Q1–Q3) or *n* (%)]	PGS 5 (*n* = 80) Value [Median (Q1–Q3) or *n* (%)]	*P* value
Age, year	54 (42–63)	51.5 (40–63.8)	54 (43–64)	52 (41–63)	51.5 (43.8–58.5)	0.443
Sex (male)	141 (31.3)	41 (28.1)	58 (27.2)	60 (36.6)	22 (27.5)	0.308
T2DM incidence	10 (2.2)	31 (21.2)	78 (36.6)	51 (31.1)	15 (18.8)	< 0.001
Previous hospital visit because of gallstone symptoms - Three visits	4 (10.8)	7 (18.9)	12 (32.4)	6 (16.2)	8 (21.8)	< 0.001
Surgery duration (minutes)	37 (30–45)	54 (45–65)	53 (45–63)	50 (40–60)	63 (52–76)	< 0.001
Laparoscopic initiation with conversion to conventional surgery	0 (0)	12 (8)	32 (15)	40 (24)	80 (100)	< 0.001
WBC count (10^3^/µl)	7.51 (6.11–8.75)	8.90 (6.96–11.30)	9.20 (7.25–11.58)	9.90 (7.15–12.36)	12.80 (9.24–14.50)	< 0.001
CRP level (mg/L)	7.00 (3.90–11.91)	15.00 (9.48–39.00)	19.00 (8.00–64.00)	22.00 (9.00–61.25)	64.00 (12.26–89.0)	< 0.001
Neutrophil count (10^3^/µl)	4.28 (3.36–5.20)	5.79 (4.14–8.70)	6.20 (4.10–9.20)	7.20 (4.22–10.10)	11.00 (5.87–12.43)	< 0.001
Platelet count (10^3^/µl)	262 (215–317)	251 (212–316)	268 (210–310)	260 (200–325)	248 (109–340)	0.748
Lymphocyte count (10^3^/µl)	2.30 (1.82–2.80)	1.70 (1.30–2.30)	1.63 (1.10–2.40)	1.32 (0.90–2.23)	0.95 (0.60–1.93)	< 0.001
Monocyte count (10^3^/µl)	0.55 (0.42–0.70)	0.62 (0.49–0.80)	0.62 (0.48–0.80)	0.59 (0.44–0.74)	0.60 (0.52–0.80)	0.0002
Albumin level (g/dL)	4.30 (4.10–4.50)	4.10 (3.70–4.40)	4.00 (3.53–4.20)	4.00 (3.60–4.30)	3.59 (3.30–4.10)	< 0.001
Neutrophil/lymphocyte ratio (NLR)	1.78 (1.46–2.38)	3.17 (2.07–6.54)	3.49 (1.75–8.39)	4.76 (2.33–10.67)	11.14 (2.49–18.96)	< 0.001
Monocyte/lymphocyte ratio (MLR)	0.23 (0.19–0.29)	0.35 (0.25–0.62)	0.36 (0.23–0.67)	0.44 (0.26–0.60)	0.56 (0.30–0.91)	< 0.001
Platelet/lymphocyte ratio (PLR)	92 (75–115)	140 (110–185)	165 (120–230)	180 (130–250)	220 (150–310)	< 0.001
CRP/albumin ratio (CAR)	3.50 (1.95–5.96)	7.50 (4.74–19.50)	9.50 (4.00–32.00)	11.00 (4.50–30.62)	32.00 (6.13–44.50)	< 0.001
Inflammatory burden index (IBI)	9 (5–20)	40 (15–90)	65 (20–150)	80 (25–220)	300 (80–900)	< 0.001
Systemic inflammation response index (SIRI)	0.97 (0.70–1.41)	1.64 (1.12–4.56)	2.18 (0.95–5.25)	2.68 (1.17–5.85)	6.60 (1.54–10.89)	< 0.001
Systemic immune-inflammation index (SII)	488 (355–617)	823 (536–1486)	928 (485–2021)	1210 (516–2602)	1779 (721–3216)	< 0.001

*Abbreviations*: *CRP* C-reactive protein, *PGS* Parkland grading scale, *WBC* White blood cell

Reference laboratory ranges: WBC: 4–10 ×10³/µL, CRP: 0–5 mg/L, neutrophils: 2–7 ×10³/µL, lymphocytes: 1–3 ×10³/µL, monocytes: 0.2–0.8 ×10³/µL, platelet count: 150–400 ×10³/µL, albumin: 3.5–5.0 g/dL, AST: 5–40 U/L, ALT: 5–41 U/L, total bilirubin: 0.2–1.2 mg/dL, direct bilirubin: 0–0.3 mg/dL, ALP: 40–130 U/L, GGT: 8–61 U/L

Univariate logistic regression analysis was performed for parameters that were significantly different among the groups. According to the results of this analysis, T2DM status (*p* < 0.001), WBC count (*p* < 0.001), CRP level (*p* < 0.001), neutrophil count (*p* < 0.001), lymphocyte count (*p* < 0.001), monocyte count (*p* < 0.001), the serum albumin concentration (*p* < 0.001), the neutrophil/lymphocyte ratio (*p* < 0.001), the platelet/lymphocyte ratio (*p* < 0.001), the CRP/albumin ratio (*p* < 0.001), the Inflammatory Burden Index (*p* < 0.001), the Systemic Inflammation Response Index (*p* < 0.001), and the Systemic Immune-Inflammation Index (*p* < 0.001) were risk factors for an increased PGS score (Table 4).

**Table 4:** Univariate logistic regression analysis for higher PGS severity

Variables	OR		%95 CI	*p* value
Age, year	0.996		0.988–1.004	0.357
T2DM incidence	0.055		0.029–0.106	< 0.001
WBC count (10^3^/µl)	1.311		1.248–1.377	< 0.001
CRP level (mg/L)	1.035		1.028–1.042	< 0.001
Neutrophils count (10^3^/µl)	1.435		1.352–1.522	< 0.001
Platelet count (10^3^/µl)	0.999		0.998–1.001	0.309
Lymphocyte count (10^3^/µl)	0.387		0.328–0.458	< 0.001
Monocyte count (10^3^/µl)	3.171		1.827–5.502	< 0.001
Albumin level (g/dL)	0.197		0.144–0.270	< 0.001
Neutrophil/lymphocyte ratio	1.407		1.321–1.498	< 0.001
Monocyte/lymphocyte ratio	13.416		7.126–25.258	< 0.001
Platelet/lymphocyte ratio	1.008		1.006–1.010	< 0.001
CRP/albumin ratio	1.071		1.057–1.085	< 0.001
Inflammatory Burden Index	1.003		1.002–1.004	< 0.001
Systemic Inflammation Response Index	1.517		1.400–1.644	< 0.001
Systemic Immune-Inflammation Index	1.001	1.001–1.002		< 0.001

*WBC* White blood cell, *CRP* C-reactive protein, *OR* Confidence interval, *OR* Odds ratio

Multivariate logistic regression analysis was performed to identify variables independently associated with higher PGS severity. T2DM status (p < 0.001), CAR (p < 0.001), NLR (p < 0.001), and IBI (p < 0.001) remained independently associated with higher PGS severity. SIRI and SII lost statistical significance in the multivariate model, likely because of collinearity among inflammatory indices (p = 0.141 for SIRI and p = 0.391 for SII) (Table 5).

**Table 5:** Multivariate logistic regression analysis for higher PGS severity

Variables	Adjusted OR	%95 CI	*p* value
T2DM status	0.012	0.006–0.024	< 0.001
CRP/albumin ratio	1.073	1.051–1.096	< 0.001
Neutrophil/lymphocyte ratio	1.875	1.531–2.295	< 0.001
Inflammatory Burden Index	0.996	0.996–0.997	< 0.001
Systemic Inflammation Response Index	0.900	0.783–1.036	0.141
Systemic Immune-Inflammation Index	1.000	0.9997–1.0008	0.391

An additional subgroup analysis was performed to specifically evaluate factors associated with high-grade PGS severity. Patients were dichotomized into PGS 1–3 and PGS 4–5 groups. In multivariate logistic regression analysis, T2DM, previous hospital visits, CAR, NLR, and IBI remained significantly associated with high-grade PGS severity (Table 6).

**Table 6:** Multivariate Logistic Regression Analysis for High-Grade PGS Severity (PGS 4–5)

Variables	Adjusted OR	%95 CI	*p* value
T2DM	1.84	1.28–2.66	0.001
Previous hospital visits	2.21	1.70–2.89	< 0.001
CAR	1.021	1.012–1.031	< 0.001
NLR	1.102	1.060–1.145	< 0.001
IBI	1.001	1.001–1.002	< 0.001

*Abbreviations*: *OR* Odds ratio, *CI* Confidence interval, *T2DM* Type 2 diabetes mellitus, *CAR* C-reactive protein-to-albumin ratio, *NLR* Neutrophil-to-lymphocyte ratio, *IBI* Inflammatory burden index

**Fig. 2:**
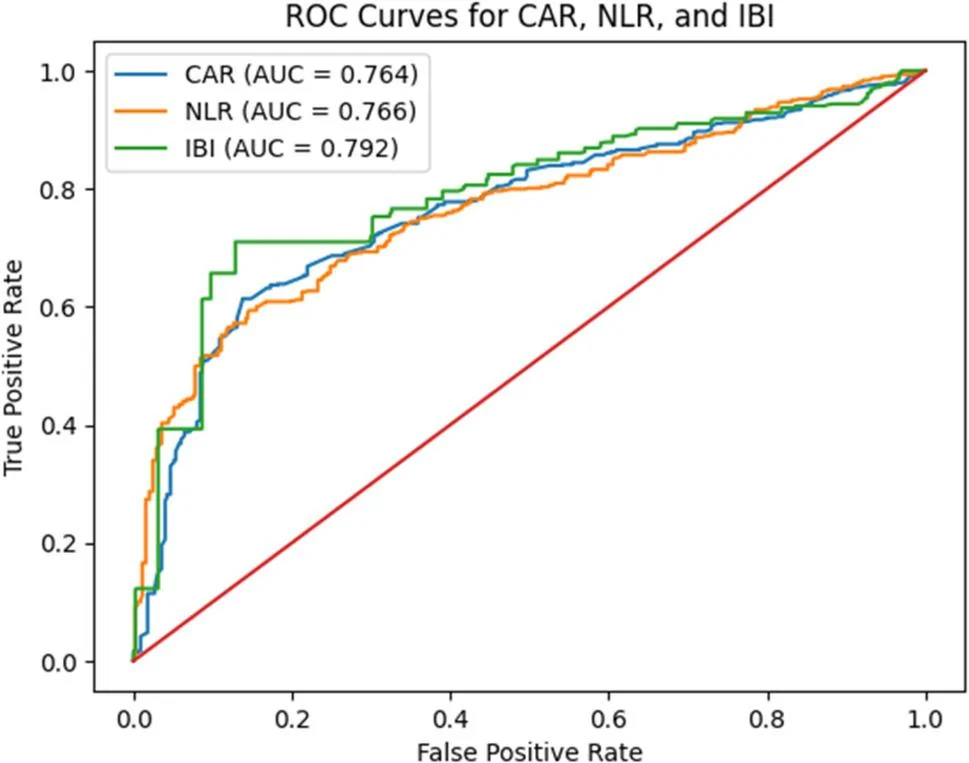
ROC curves for the CAR, NLR and IBI

Receiver operating characteristic (ROC) curve analysis was performed for CAR, NLR, and IBI, which were identified as variables independently associated with higher PGS severity in multivariate logistic regression analysis. According to the ROC analysis, the cut-off value was 7.18 for CAR, 3.03 for NLR, and 27.46 for IBI. Among the evaluated inflammatory indices, IBI demonstrated the highest AUC, sensitivity, and specificity values (Table 7; Fig. 2).

**Table 7:** ROC analysis of inflammatory indices associated with higher PGS severity

Variables	AUC	Cut-off	Sensitivity	Specificity
CRP/albumin ratio	0.764	7.18	61.4%	86.3%
Neutrophil/lymphocyte ratio	0.766	3.03	59.4%	85.4%
Inflammatory Burden Index	0.792	27.46	70.9%	87.1%

*Abbreviations*: *AUC* Area under the receiver operating characteristic curve, *CRP* C-reactive protein

## Discussion

Our study demonstrated a significant association between the Parkland Grading Scale (PGS) and several systemic inflammatory indices in patients undergoing elective laparoscopic cholecystectomy for gallstone disease. Among these indices, IBI, NLR, CAR, SIRI, and SII increased progressively with increasing PGS grades, suggesting that greater systemic inflammatory burden is associated with higher intraoperative inflammatory severity and operative complexity. In addition, higher PGS grades were associated with longer operative duration and increased conversion rates to open surgery.

Owing to the variability of intraoperative findings, laparoscopic cholecystectomy remains one of the more technically variable procedures in general surgery. This variability is influenced not only by anatomical factors but also by inflammation, fibrosis, adhesions, and distortion of the dissection planes within the Calot triangle [18]. Because of these factors, several intraoperative grading systems have been developed to standardize the assessment of operative severity [19, 20]. One of these systems, the PGS, was first introduced by Madni et al. [14] in 2018 as a simple and practical grading system based on inflammatory and anatomical intraoperative findings. In a subsequent prospective validation study, Madni et al. [15] reported that the PGS demonstrated high reliability and was significantly associated with laparoscopic cholecystectomy outcomes. Similarly, the PGS was reported to perform better than the Tokyo Guidelines Grading System in reflecting postoperative treatment requirements, particularly in severe cases [21]. Liu et al. [22] also demonstrated that increasing PGS grades were associated with greater operative complexity during laparoscopic cholecystectomy.

In the present study, operative complexity was primarily reflected by operative duration and conversion from laparoscopic to open surgery. Durmuş et al. [23] reported that conversion rates were significantly higher in patients with PGS 3–5 compared with those with PGS 1–2. Similarly, in our cohort, the PGS 5 group demonstrated the highest conversion rate. This finding may reflect severe chronic inflammatory changes, fibrosis, dense adhesions, and distorted anatomy encountered in these patients. In addition, institutional surgical decision-making and lower thresholds for conversion in technically challenging cases may also have contributed to the relatively high conversion rates observed in the present study. As PGS severity increased, operative duration also increased significantly. Madni et al. [14] similarly demonstrated that higher PGS grades were associated with prolonged operative time.

Several inflammatory biomarkers, including the neutrophil-to-lymphocyte ratio (NLR), platelet-to-lymphocyte ratio, and systemic immune-inflammation index, have previously been investigated as markers of inflammatory severity in acute calculous cholecystitis [4, 24]. Fındık et al. [25] reported that NLR, PLR, and SII were significantly associated with disease severity in acute cholecystitis. In our study, NLR was also significantly associated with increasing PGS severity; however, the cut-off value identified in our cohort (3.03) was lower than that reported in acute cholecystitis studies. This difference may be explained by the elective nature of our cohort, which predominantly reflected chronic or stabilized inflammatory processes rather than active severe acute inflammation. Similarly, Kler et al. [26] demonstrated that NLR values were substantially higher in acute and severe cholecystitis, supporting the interpretation that inflammatory marker levels vary according to the intensity and timing of inflammation. One important aspect of this study is that only elective cholecystectomy cases performed under stabilized clinical conditions were included. Although inflammatory markers may be lower in elective settings than in acute cholecystitis, chronic inflammation, recurrent biliary attacks, fibrosis, and adhesions may still contribute to higher PGS severity and operative complexity. Therefore, the observed associations may reflect chronic inflammatory burden rather than active acute inflammation. Residual inflammatory marker elevation in some high-grade PGS patients may reflect recurrent biliary attacks, chronic inflammation, fibrosis, or recent conservatively managed inflammatory episodes prior to elective surgery (reviewer 4 − 2).Severe pathological findings such as perforation or necrosis may be less frequently encountered in elective settings; however, advanced chronic inflammatory changes, fibrosis, and distorted anatomy may still result in high PGS grades.

CRP is a well-established inflammatory marker, whereas albumin is a negative acute-phase reactant associated with nutritional and systemic inflammatory status. Güneş et al. [27] reported that the CRP/albumin ratio (CAR) was associated with severe acute calculous cholecystitis and perioperative complications. In our study, higher CAR values were significantly associated with increasing PGS severity and operative complexity.

The Inflammatory Burden Index (IBI) was originally developed as a marker reflecting systemic inflammatory burden and prognosis in various malignancies [6]. Interest in IBI has increased because its components—CRP, neutrophil count, and lymphocyte count—reflect different aspects of systemic inflammatory response [28, 29]. To our knowledge, there are currently no studies evaluating the relationship between IBI and PGS severity in laparoscopic cholecystectomy patients. In the present study, IBI demonstrated the strongest association with increasing PGS grades among the evaluated inflammatory indices. Rather than serving as a definitive predictive marker, IBI may reflect the degree of inflammatory and anatomical severity encountered intraoperatively during laparoscopic cholecystectomy. The observed association likely results from the combined inflammatory information provided by CRP, neutrophil count, and lymphocyte count, which together may better represent chronic inflammatory burden and operative complexity. Additional subgroup analysis focusing on PGS 4–5 cases demonstrated that inflammatory indices, particularly IBI, NLR, and CAR, remained significantly associated with high-grade PGS severity after adjustment for clinical variables. These findings suggest that systemic inflammatory burden may be more pronounced in patients with severe intraoperative inflammatory and anatomical changes associated with technically difficult laparoscopic cholecystectomy.

Recent advances in artificial intelligence (AI) have introduced new possibilities for real-time intraoperative anatomical recognition during laparoscopic cholecystectomy. AI-assisted image recognition systems have shown promising results in identifying critical anatomical landmarks and supporting safer dissection strategies. Kehagias et al. recently reported that AI-based models demonstrated clinically feasible performance for intraoperative landmark detection during laparoscopic cholecystectomy; however, their sensitivity decreased in cases with severe inflammation and advanced cholecystitis [30]. These findings are particularly relevant to higher PGS grades, where fibrosis, adhesions, and distorted anatomy substantially increase operative complexity. Future integration of inflammatory indices, intraoperative grading systems such as PGS, and AI-assisted surgical guidance may contribute to safer management of technically difficult cholecystectomy cases.

### Limitations and strengths of the study

This study has several limitations. First, because of its retrospective cross-sectional design, causal relationships cannot be established, and the findings should be interpreted as associations rather than definitive predictive implications. Second, the single-center nature of the study may limit the generalizability of the results to different patient populations and surgical settings. In addition, operative complexity during laparoscopic cholecystectomy is multifactorial and may be influenced by several clinical and anatomical variables beyond inflammatory burden. Factors such as body mass index (BMI), previous abdominal surgery, particularly prior upper abdominal incisions, fibrosis, adhesions, and anatomical variations related to the gallbladder–liver relationship may significantly affect operative complexity and were not comprehensively evaluated in the present study. Another limitation is that laparoscopic cholecystectomy procedures were performed by multiple surgeons, which may have introduced variability in operative decision-making and conversion thresholds. In addition, PGS grading was retrospectively determined from operative reports rather than prospectively assigned intraoperatively in a standardized manner, which may have introduced some degree of grading variability. Because inflammatory indices and operative severity were evaluated within the same retrospective cohort, the observed relationships should be interpreted as associations rather than evidence of direct causality or definitive predictive effects. In addition, the interval between the last symptomatic biliary episode and elective surgery was not consistently available in all patients because of the retrospective design. Despite these limitations, the study includes a relatively large patient cohort and provides novel data regarding the association between inflammatory indices and PGS severity.

## Conclusions

Based on the PGS (Processive Surgical Grid), which provides an intraoperative assessment of surgical severity during laparoscopic cholecystectomy, preoperative inflammatory indices such as CRP/albumin ratio, neutrophil/lymphocyte ratio, and inflammatory load index were found to be significantly associated with higher PGS scores and increased surgical difficulty. The determined threshold values (7.18 for CAR, 3.03 for NLR, and 27.46 for IBI) may be useful for predicting increased surgical severity in patients undergoing elective laparoscopic cholecystectomy. In this context, systemic inflammatory load may reflect the degree of intraoperative inflammatory severity encountered during surgery.

## Data Availability

The datasets generated and/or analysed during the current study are not publicly available due institutional regulations and ethical restrictions related to patient confidentiality, but are available from the corresponding author on reasonable request. So, all data access requests will be evaluated in accordance with institutional data protection policies.

## Abbreviations

LC: Laparoscopic cholecystectomy
IBI: Inflammatory Burden Index
CRP: C-reactive protein
SIRI: Systemic Inflammation Response Index
SII: Systemic Immune-Inflammation Index
PGS: Parkland grading scale
NLR: Neutrophil/lymphocyte ratio
MLR: Monocyte/lymphocyte ratio
PLR: Platelet/lymphocyte ratio
CAR: CRP/albumin ratio
TGGS: Tokyo Guidelines Grading System
